# Capnocytophaga canimorsus infection presenting with complete splenic infarction and thrombotic thrombocytopenic purpura: a case report

**DOI:** 10.1186/1756-0500-5-695

**Published:** 2012-12-26

**Authors:** Michal Brichacek, Peter Blake, Raymond Kao

**Affiliations:** 1 Critical Care Trauma Centre, London Health Sciences Center, 800 Commissioners Road East, London, Ontario N6A 5W9, Canada; 2 Department of Nephrology, London Health Sciences Center, 800 Commissioners Road East, London, Ontario N6A 5W9, Canada; 3 Department of National Defense, Canadian Forces Health Services, 1745 Alta Vista Drive, Ottawa, Ontario K1A 0K6, Canada

**Keywords:** Capnocytophaga canimorsus, Thrombotic thrombocytopenic purpura, Splenic infarction, Dog bite

## Abstract

**Background:**

Animal bites are typically harmless, but in rare cases infections introduced by such bites can be fatal. *Capnocytophaga canimorsus*, found in the normal oral flora of dogs, has the potential to cause conditions ranging from minor cellulitis to fatal sepsis. The tendency of *C*. *canimorsus* infections to present with varied symptoms, the organism’s fastidious nature, and difficulty of culturing make this a challenging diagnosis. Rarely, bacterial cytotoxins such as those produced by *C*. *canimorsus* may act as causative agents of TTP, further complicating the diagnosis. Early recognition is crucial for survival, and the variability of presentation must be appreciated. We present the first known case of *C*. *canimorsus* infection resulting in TTP that initially presented as splenic infarction.

**Case presentation:**

72-year-old Caucasian male presented with a four-day history of abdominal pain, nausea, vomiting, diarrhea, and intermittent confusion. On presentation, vital signs were stable and the patient was afebrile. Physical examination was unremarkable apart from petechiae on the inner left thigh, and extreme diffuse abdominal pain to palpation and percussion along with positive rebound tenderness. Initial investigations revealed leukocytosis with left shift and thrombocytopenia, but normal liver enzymes, cardiac enzymes, lipase, INR and PTT. Abdominal CT demonstrated a non-enhancing spleen and hemoperitoneum, suggesting complete splenic infarction. Although the patient remained afebrile, he continued deteriorating over the next two days with worsening thrombocytopenia. After becoming febrile, he developed microangiopathic hemolytic anemia and hemodynamic instability, and soon after was intubated due to hypoxic respiratory failure and decreased consciousness. Plasma exchange was initiated but subsequently stopped when positive blood cultures grew a gram-negative organism. The patient progressively improved following therapy with piperacillin-tazobactam, which was switched to imipenem, then meropenem when Capnocytophaga was identified.

**Conclusions:**

There is a common misconception amongst practitioners that the presence of systemic infection excludes the possibility of TTP and vice versa. This case emphasizes that TTP may occur secondary to a systemic infection, thereby allowing the two processes to coexist. It is important to maintain a wide differential when considering the diagnosis of either TTP or *C*. *canimorsus* infection since delays in treatment may have fatal consequences.

## Background

Animal bites are a common occurrence and usually involve dogs and cats, with the majority being minor and requiring no medical attention. However, in certain rare cases, an infection introduced by such a bite can be fatal. The genus *Capnocytophaga* consists of seven species, with only *Capnocytophaga cynodegmi* and *Capnocytophaga canimorsus* being considered human pathogens, both of which can be found in the normal oral flora of dogs and less commonly that of cats [1]. *C*. *canimorsus* infections have been specifically linked to dog and cat bites or scratches, and close animal contact such as licking of wounds [2]. It is estimated that between 8% and 26% of canines may have *C*. *canimorsus* as part of their normal flora, although unpublished reports have reported higher percentages [1,2].

Although *C*. *canimorsus* is an uncommon low virulence pathogen in humans, it has the potential to cause conditions ranging from a minor cellulitis to serious conditions such as endocarditis, meningitis, sepsis, disseminated intravascular coagulopathy (DIC), gangrene, acute renal failure, and respiratory failure [3,4]. The tendency of *C*. *canimorsus* infections to present with a wide range of varied signs and symptoms makes this a challenging diagnosis. Identification of the organism is further complicated by its fastidious nature and difficulty to culture. Asplenic individuals are at particular risk for infection in 33% of reported cases and have a thirty to sixty-fold increased risk of succumbing to fatal sepsis with a 70% mortality rate [5]. In this patient subset an overwhelming infection can progress to multiple organ system failure and death within twenty-four hours, making early recognition is critical to survival. In rare cases, bacterial cytotoxins such as those produced by *C*. *canimorsus* may act as causative agents of TTP [6]. Herein we present the first known case of *C*. *canimorsus* infection resulting in TTP, which initially presented as a splenic infarction.

## Case presentation

Mr. R.E. is a 72-year-old man who presented to the emergency department with a four-day history of abdominal pain, nausea, vomiting and diarrhea, which at one point progressed to confusion and slurred speech. Past medical history was most significant for a fall four months previously that resulted in fracture of several ribs on his left side. On presentation he was alert, oriented, and hemodynamically stable with: temperature 36.7°C, BP 143/83 mmHg, HR 85 beats per minute, RR 20 breaths per minute, oxygen saturation at 96% on room air. He denied shortness of breath, fever, chills, night sweats, or weight loss. Cardiac, pulmonary, and neurological examinations were unremarkable. Abdominal examination revealed extreme diffuse pain to palpation and percussion, positive rebound tenderness, but no evidence of hepatosplenomegaly. Mucous membranes were dry, and petechiae were observed along the inner left thigh.

**Figure 1:**
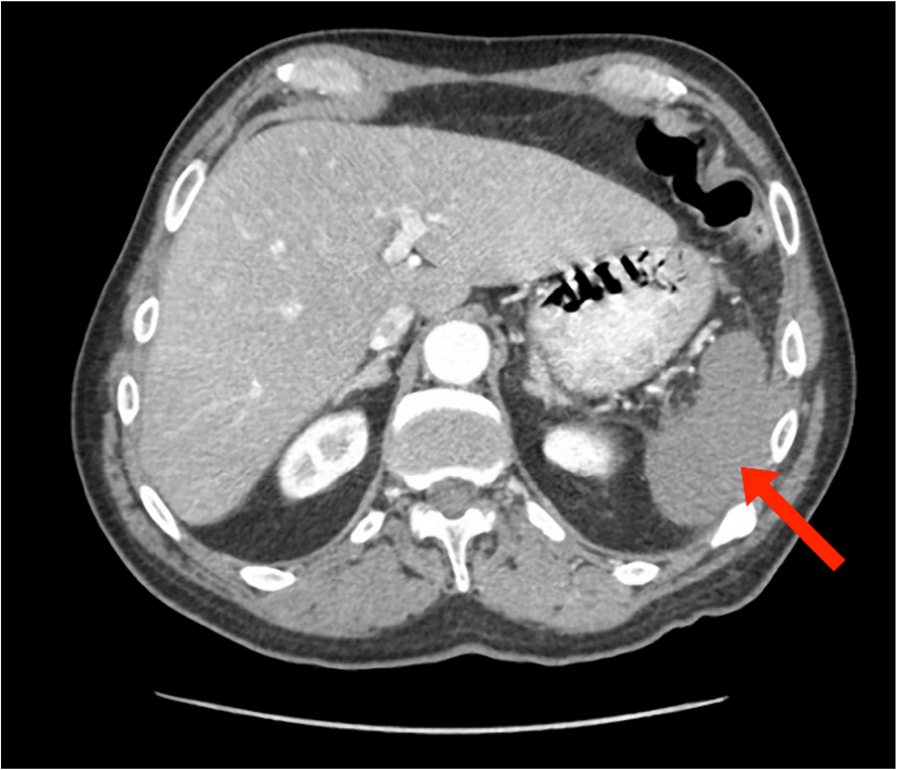
CT-scan of the abdomen obtained on presentation showed complete non-enhancement of the spleen (arrow) supporting complete splenic infarction.

Initially laboratory investigations revealed: hemoglobin (Hb) 138 g/L, white blood cell count (WBC) 14.7×10^9^/L, neutrophil count 13×10^9^/L, platelets 28×10^9^/L, sodium 132 mmol/L, chloride 95 mmol/L, potassium 4.2 mmol/L, bicarbonate 27 mmol/L, BUN 18.6 mmol/L, plasma creatinine 112 μmol/L, total bilirubin 22.2 μmol/L, international normalized ratio (INR) 1.1, prothrombin time (PTT) 30 seconds, aspartate aminotransferase (AST) 95 U/L, alkaline phosphatase 57 U/L, serum creatine kinase (CK) 807 U/L, troponin-T < 0.01 μg/L, lactate dehydrogenase (LDH) 282 U/L, serum lipase 13 U/L and total amylase 58 U/L. Urinalysis was positive for blood with 20 to 30 erythrocytes per high power field, protein 1.0 g/L and pH of 6.5. A CT of the abdomen with contrast showed the entire spleen to be non-enhancing suggesting complete infarction, which was suspected to be caused by spontaneous embolization of the splenic artery at the hilum (Figure 1). No definite active vascular extravasation was seen, however there was soft tissue stranding, a small amount of high attenuating fluid in the perisplenic fat and along the right paracolic gutter, and a small to moderate volume of hemoperitoneum in the pelvis.

Although an atypical presentation, the initial concern was a microangiopathic disorder such as thrombocytopenic thrombotic purpura (TTP), hemolytic uremic syndrome (HUS), or idiopathic thrombocytopenic purpura (ITP). Additional investigations for other vasculitic or autoimmune causes showed: C3 complement level 0.55 g/L (normal 0.66 to 1.68 g/L), C4 complement level 0.07 g/L (normal 0.10 to 0.40 g/L), antinuclear antibody (ANA) very weakly positive. pANCA, cANCA and anti-glomerular basement membrane antibodies were all negative. Direct and indirect Coombs tests, as well as anti-phospholipid IgM and IgG were likewise all negative. Five units of platelets were transfused but it did not increase the platelet count. Thirty-six hours following presentation the hemoglobin remained stable (125 g/L), WBC decreased to 7.5×10^9^/L, erythrocyte sedimentation rate (ESR) was elevated at 38 mm/h, and C reaction protein (CRP) was elevated at 346.3 mg/L. The peripheral blood smear showed reactive granulocytosis but no evidence of hemolysis, and the patient remained afebrile.

Forty-eight hours after presentation the patient’s condition began to deteriorate with: T 37.8°C, HR 110, RR 26, oxygen saturation at 92% on room air. The patient had high fever, chills, rigor and low back pain, but physical examination was unremarkable. Platelet count further decreased to less than 10×10^9^/L, hemoglobin decreased to 109 g/L, serum LDH increased to 306 U/L, and total bilirubin increased to 45.7 μmol/L along with an increase in creatinine from 74 to 102 μmol/L. There was no evidence of disseminated intravascular coagulopathy (DIC) given a normal Klaus fibrinogen level at 4.30 g/L, INR 1.1, and PTT 26 seconds.

On day three of admission, peripheral blood smears showed some evidence of mild schistiocytes, suggesting the presence of microangiopathic hemolytic anemia (MAHA). Pulmonary condition began to deteriorate, with increased requirement to 6 liters of oxygen per minute producing an oxygen saturation of 88 to 90%. The patient became hemodynamically unstable with a BP 95/58 and experienced decreased level of consciousness. Chest X-ray showed bilateral pulmonary infiltrates with evidence of pulmonary edema. He was transferred to the intensive care unit and intubated due to hypoxic respiratory failure and decreased level consciousness. The nephrology service was consulted and plasma exchange therapy was empirically initiated on the suspicion for TTP as the etiology of his deterioration. Following initiation of plasma exchange, blood culture results obtained on admission to the hospital were found to contain gram-negative rods, and piperacillin-tazobactam 4.5 grams IV Q8H was initiated. Two subsequent blood cultures both showed evidence of an unknown gram-negative organism and the patient remained febrile with temperature 39.5°C. Cardiac echocardiogram did not reveal any valvular vegetation and a head CT scan was also unremarkable. Plasma exchange was stopped on day five of admission due to failure of platelet count to increase with plasma exchange therapy, the presence of gram negative blood cultures, and the degree of thrombocytopenia being largely out of proportion to the level of LDH rise.

**Figure 2:**
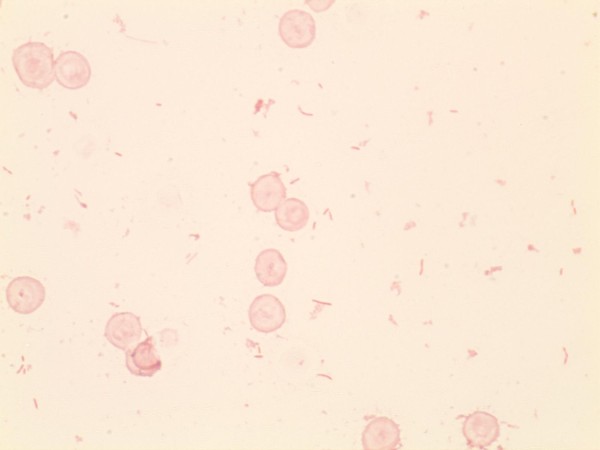
**Gram stain prepared from a positive blood culture vial demonstrating the presence of red blood cells together with the presence of *C*. *canimorsus.***

**Figure 3:**
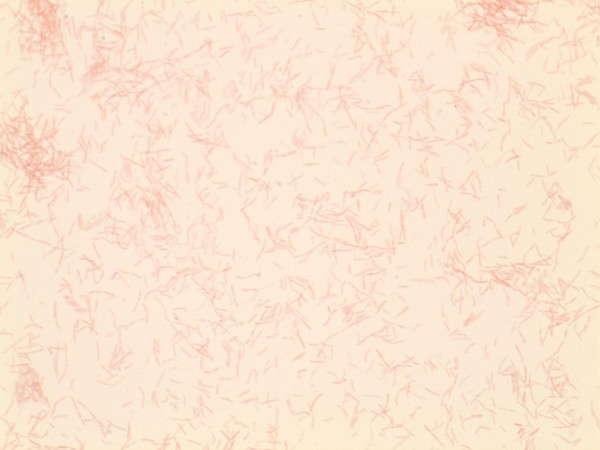
**Gram stain prepared from a culture plate shows the presence of a fastidious, facultatively anaerobic, fusiform organism that was determined to be *C*. *canimorsus.***

On day six of admission, the microbiology laboratory reported that the gram-negative organism identified to be a capnocytophaga species based on its fastidious growth and morphology on gram stain (Figures 2 and 3). Antibiotic therapy was switched to imipenem 500 mg IV Q6H given reports of some capnocytophaga species being resistant to certain beta-lactams [7]. Subsequently, on day seven of admission the platelet count increased to 60×10^9^/L, but the patient continued to be febrile with a temperature of 38.0°C and WBC count continued to increase to 22.5×10^9^/L. Due to his persistent confusion and decreased level of consciousness, antibiotic therapy was changed to meropenem 2 grams IV Q6H to decrease the risk of imipenem induced seizure activity. In an attempt to identify the cause of the splenic infarction a trans-esophageal echo was performed, but it did not demonstrate valvular vegetations.

The patient’s condition began to improve clinically with meropenem. Fever gradually resolved, platelet count continued to increase, and WBC count began to normalize. Following extubation the patient’s neurological status continued to improve. On further questioning, the patient reported that several days prior to his presentation to the hospital he sustained a cut to his hand while performing automobile repairs. He reports that shortly after sustaining this cut that his dog licked his wound. The patient also reported that he plays with his dog in the evenings on a regular basis and often receives minor bites or scratches to his hands.

The patient was discharged home from the hospital on day twenty-one of his admission with no further antibiotic therapy. On discharge his WBC count was 14.2×10^9^/L and platelets were 295×10^9^/L. Blood culture samples were sent to the Central Public Health Laboratory, Toronto, Canada and through gene sequencing the organism *C*. *canimorsus* was isolated.

## Discussion

*C*. *canimorsus*, formally called dysgonic fermenter 2 (DF-2) is a gram-negative, capnophylic (carbon dioxide loving), non-spore forming, fastidious bacillus [3]. These properties make it a difficult organism to culture, as it requires blood agar for optimal growth and increased levels of carbon dioxide, and even then it displays slow and minimal growth [3]. The differential diagnosis of *C*. *canimorsus* from blood films is possible when gram-negative bacilli are seen within the neutrophils, however definitive diagnosis is not possible until at least five days of culture in laboratory, which was consistent with our case [4,8]. Such difficulty has made confirmatory diagnosis with broad range polymerase chain reaction followed by DNA sequencing more commonplace [1]. Although it is a common organism in the flora of dogs, it has the potential to cause serious but rare infection in humans. The infection is more likely to occur in men, especially in those with conditions such as immunocompromise, asplenia, alcoholism, chronic lung disease, and hematological malignancies [8].

We are not able to conclude with certainty whether the patient was in a state of immunocompromise that made him susceptible to infection. Unfortunately, quantitative immunoglobulins or the phagocytic capability of polymorphic neutrophils were not part of the laboratory investigations. However, we know that the patient was able to mount a white blood cell response to the inflammatory/infectious process, and we also know that the patient responded positively to antibiotic therapy. These points suggest that the patient was likely not immunocompromised, but we cannot be certain of this without a measurement of the phagocytic capability of the neutrophils. Other vasculitic and autoimmune markers however were examined and all found to be negative.

Our patient likely became infected with *C*. *canimorsus* when his canine companion licked his minor open wound. This wound was so minor that it was not noted on admission, and at no point in the patient’s hospitalization did this wound become swollen, painful and erythematous. This case highlights the importance of considering zoonotic infections in patients presenting with atypical problems who may have even simple contact with household pets. Furthermore, it also demonstrates the importance of maintaining a wide differential diagnosis when encountering a patient with an atypical presentation of TTP, as this may be occurring secondary to another disease process. An infectious process was considered initially, but the peripheral blood smear demonstrated schistiocytes with associated thrombocytopenia, mild proteinuria, confusion and fever, directing the presumptive diagnosis of TTP. The cause of the seemingly idiopathic splenic artery occlusion resulting in complete splenic infarction was most likely the result of the thrombotic process from the presumptive diagnosis of TTP. Although the patient did report a history of left flank trauma four months prior to admission to hospital in which he sustained several broken ribs, this would have resulted in a different mechanism of splenic infarction. The CT scan was reviewed with an interventional radiologist at our institution who interpreted that there did not appear to be a single embolus of the splenic artery that resulted in infarction, but rather the presence of multiple emboli immediately after the splenic artery enters the spleen resulting in complete infarction. The presence of a pseudoaneurysm that may have formed as a result of the traumatic insult several months previously was also ruled out, making a microangiopathic cause more likely.

The patient initially had the developing features suggestive of TPP, meeting four symptoms of the classical pentad of thrombocytopenic purpura: thrombocytopenia, fluctuating neurological symptoms, renal failure, and fever [9]. It is imperative to note that INR and PTT remained normal, which is a crucial distinguishing point from DIC. Microangiopathic hemolytic anemia followed as evidenced by decreased hemoglobin, elevated bilirubin and LDH, and presence of schistiocytes on blood smear. Although TTP was the working diagnosis, it was later excluded in the presence of positive blood cultures and a lack of response to platelet exchange therapy. However, response to platelet exchange therapy is not universal, with up to 20% of individuals with TTP/HUS being non-responders to plasma exchange therapy [10]. In retrospect, the patient likely had an atypical presentation of TTP secondary to *C*. *canimorsus* sepsis. Bacterial cytotoxins may act as causative agents in TTP, with the most common causative agent being *Escherichia coli*, but *Shigella dysenteriae*, *Salmonella typhi*, and *Campylobacter jejuni* have all been implicated [6].

Production of cytotoxin by *C*. *canimorsus* has also been established, demonstrating toxin facilitated destruction of phagocytic and other immune cells during active disease [6,11,12]. Interestingly, the related organism *Capnocytophaga cynodegmi* does not produce cytotoxin, which may suggest why it is not as significant of a human pathogen as *C*. *canimorsus*[6]. Further virulence studies of *C*. *canimorsus* have found that all dog strains possess a sialidase, which inhibits bactericidal activity of macrophages and also block release of nitric oxide by LPS-stimulated macrophages [12]. Although all strains possessed sialidase, only 60% of these blocked the killing of phagocytosed *Escherichia coli* by macrophages and an even lower 6.5% blocked the release of nitric oxide by LPS-challenged macrophages, which may account for the difference in presentations and severity amongst *C*. *canimorsus* infections [12].

The lack of response to plasma exchange therapy certainly does not disqualify the diagnosis of TTP, but rather may suggest the possibility of a secondary cause of TTP [13]. It has been well established that certain systemic infections may in fact trigger the onset of an acute episode of TTP, meaning that a patient may have both conditions concurrently [13]. In such cases, it is not uncommon for patients to be non-responsive to plasma exchange therapy as a result of their ongoing systemic infection [13]. The diagnosis of disseminated intravascular coagulopathy (DIC) is also a possibility that can cause thrombocytopenia and microangiopathic hemolytic anemia, but it is also associated with prolongation of both the INR and PTT, which were not observed in this patient. It is also important to remember that patients with TTP often do not experience all the features of the classical pentad [11]. This may be suggestive of a less severe variant of TTP secondary to an infective process, and may have been the key to the patient’s survival.

Although rare, there have been reports in the literature of *C*. *canimorsus* infection producing secondary TTP [13,14]. In one case, the patient was treated with plasma exchange and a single dose of antibiotics, while in another the patient was treated solely with imipenem, yet both patients were found to improve within a matter of days [14]. In another case, *C*. *canimorsus* infection presented as a TTP like syndrome with all the symptoms of the classical pentad present, but the patient likewise had an abnormal clotting screen showing mildly elevated INR and PTT indicating an element of DIC. In our case, the patient received plasma exchange therapy in addition to treatment with piperacillin-tazobactam, followed by imipenem, and then meropenem, and likewise recovered in a period of several days.

The von Willebrand factor cleaving protease, ADAMTS13, may act as a helpful marker in the diagnosis of classic immune-mediated TTP [13]. The ADAMTS13 test is not performed at our facility, and must instead be sent to an outside laboratory at a significant cost. Since results would not be available for several weeks and would not influence our acute decision making regarding treatment, it was felt this test was unnecessary. Even if results were available earlier, they would not have influenced our management as a deficiency of ADAMTS13 activity or the presence of an ADAMTS13 inhibitor would not have excluded the possibility of a systemic infection resulting in TTP. Furthermore, there have been reports of typical cases of TTP that do not present with low ADAMTS13 activity [15].

Given the variability in presentation seen in TTP caused by *C*. *canimorsus* and previous case reports describing *C*. *canimorsus* septicemia as a cause of TTP/HUS, it is certainly a reasonable explanation for our patient’s presentation and further establishes the organism as a potential cause of TTP/HUS [14]. Although the mechanism of splenic infarction was confounded by the patient’s recent trauma, it is likely that TTP secondary to *C*. *canimorsus* caused thrombotic occlusion of the splenic artery resulting in complete splenic infarction. Ironically, it is possible that this infarction was in fact helpful with patient’s TTP by rendering him asplenic. Plasma exchange therapy remains the first-line treatment for TTP, however for patients refractory to plasma exchange or for those with relapsing disease requiring frequent plasma exchange therapy to maintain remission, the optimal treatment includes immunosuppressant medications or splenectomy [16]. While sepsis may cause thrombocytopenia and coagulopathy, this would occur in the context of DIC and would affect both INR and PTT, both of which were normal in our patient. Endocarditis was likewise considered to be a possible source of an embolus, but trans-esophageal echocardiogram was unequivocally normal.

## Conclusion

The difficult microbiological identification of *C*. *canimorsus* is associated with high mortality because it has the ability to present with a wide range of signs and symptoms, which would delay appropriate medical treatment. This case is of particular interest because it demonstrates a peculiar presentation of *C*. *canimorsus* infection in an initially afebrile patient presenting with acute splenic infarction with isolated thrombocytopenia, followed by a secondary insult of TTP. This unusual constellation of signs and symptoms initially misled our diagnosis, as it was not entirely suggestive of either TTP or an infective process. This emphasizes the importance of maintaining a wide differential diagnosis when considering the diagnosis of TTP and realizing that TTP may in fact occur secondary to a systemic infection, in which case the two processes may exist concurrently. There is a common misconception amongst practitioners that the presence of systemic infection excludes the possibility of TTP and vice versa, which clearly was not the case in our patient.

## Consent

Written informed consent was obtained from the patient for publication of this case report and accompanying images. A copy of the written consent is available for review by the Editor-in-Chief of this journal.

## Competing interests

The authors of this case report declare that they have no competing interests.

## Authors’ contributions

MB wrote the bulk of the manuscript and performed background research on the subject. RK was the attending critical care physician involved in the case and was a major contributor in writing and editing the manuscript. PB was the nephrologist involved with the case and was also a contributor in writing and editing the manuscript. All authors read and approved the final manuscript.

## Preface

While *C*. *canimorsus* infection has been found to result in thrombocytopenic purpura in very rare cases, we present the first known case of such a process developing gradually and initially presenting as a complete splenic infarction. This report will further our knowledge of the possible varied presentation of *C*. *canimorsus* infection, which is crucial to adequate management of this rare and often misdiagnosed condition. This report is not of interest to only one medial specialty, but rather is applicable to a broader audience in the areas such as, but not limited to: nephrology, hematology, infectious disease, family medicine, emergency medicine, and critical care medicine.
